# Identifying Gingival Pigmentation Patterns and Skin Color and Its Co-relation With Serum Ferritin Levels in Thalassemic Patients

**DOI:** 10.7759/cureus.28015

**Published:** 2022-08-14

**Authors:** Shreya Gajjar, Harjot Kaur, Gaurav Girdhar, Ashish Kaur, Chandni Patel, Rupal Mehta, Sushmita Bhakkand, Tanvi Hirani, Surabhi Joshi, Mohammed Irfan, Wan Farizatul Shima Binti Wan Ahmad Fakuradzi, Susmita Sinha, Mainul Haque, Santosh Kumar

**Affiliations:** 1 Periodontology, Karnavati School of Dentistry, Karnavati University, Gandhinagar, IND; 2 Periodontology, Karnavati University, Gandhinagar, IND; 3 Periodontology, AMC (Ahmedabad Municipal Corporation) Dental College and Hospital, Ahmedabad, IND; 4 Forensic Dentistry, Federal University of Pelotas, Pelotas, BRA; 5 Community Medicine, Faculty of Medicine and Defence Health, Universiti Pertahanan Nasional Malaysia, Kuala Lumpur, MYS; 6 Physiology, Khulna City Medical College and Hospital, Khulna, BGD; 7 Pharmacology and Therapeutics, National Defence University of Malaysia, Kuala Lumpur, MYS; 8 Periodontology and Implantology, Karnavati University, Gandhinagar, IND

**Keywords:** gum color, thalassemia, serum ferritin, iron overload, gingival pigmentation

## Abstract

Background: Patients with β-thalassemia major (β-TM), a genetic issue due to hemoglobin (Hb) synthesis disorder, require life-long erythrocyte transfusion. The purpose of this study is to evaluate and compare gingival pigmentation and skin color with serum ferritin levels of patients with β-TM, using the Dummett's oral pigmentation index (DOPI) and Fitzpatrick skin scale, respectively.

Methods: A total of 100 patients were monitored at a thalassemia care center. Each patient's gingival pigmentation and skin color were matched with DOPI and the skin scale under natural light. Serum ferritin levels, the interval of blood transfusions, and iron chelation medications were studied. A gingival pigmentation score and skin color type were compared with the serum ferritin.

Results: A significant correlation was found between age, serum ferritin, pigmentation score, and skin color, which means as serum ferritin level increases, gingival pigmentation score increases, and skin color darkens.

Conclusion: This study evaluated the correlation between gingival pigmentation and skin color with serum ferritin levels and established gingival pigmentation as a sign of iron deposition in β-TM patients. This is the simplest and least invasive method for evaluating serum ferritin level parameters in β-TM patients.

## Introduction

Thalassemia is a usual cause of microcytic hypochromic anemia, which occurs because of diminishing or lack of synthesis of the globin chain in hemoglobin [1]. This is dissimilar from other hemoglobinopathies, such as sickle cell disease, which are anatomic defects of hemoglobin [2]. β-thalassemia is a genetic alteration of the beta-globin gene, causing a reduction in beta-globin chain production [3]. The maximum prevalence of beta-thalassemia is in people of Mediterranean, Middle Eastern, and Asian descent [4]. Around 200 different genetic mutations have been identified, making the disease widely variable, genotypically and phenotypically [5]. β-thalassemia major (β-TM) is a hereditary type of hemolytic anemia that was first described in 1925 by Thomas Benton Cooley, an American pediatrician and hematologist [6], so it is also called Cooley's anemia. Studies have shown that the overall prevalence of β-thalassemia in India is 3-4%, with an estimated 8,000-10,000 new births with the significant disease each year [7].

Patients with β-TM require regular erythrocyte transfusion at specific intervals [8]. Ineffective erythropoiesis and increased gastrointestinal iron absorption lead to iron overload in the body, primarily in the form of ferritin [9,10]. A unit of red blood cells transfused contains approximately 250 mg of iron, while the body cannot excrete more than 1 mg of iron per day [11]. There are various methods to check iron overload deposition, such as liver biopsy, skin biopsy, magnetic resonance imaging (MRI) of the heart and liver, and investigating serum ferritin and non-transferrin bound iron [12]. The most commonly used method is the measurement of serum (plasma) ferritin levels [13].

Dermatological manifestations of patients with β-TM are darkened skin color and gingival hyperpigmentation due to iron overloading [5]. It is seen that there is a direct correlation between skin color and body iron levels in patients with β-TM [14]. Oral manifestations of thalassemia are malocclusion, high caries index, mucosal pallor, severe gingivitis, inflammation of salivary glands, thin mandibular cortex, multiple diastemas, spike-shaped and short roots, taurodontism, and dark-colored gingiva [15]. Excessive deposition of melanin in basal and suprabasal layers of epithelium results in gingival hyperpigmentation [16]. Studies have shown that cause of the dark-colored gingiva is high ferritin levels in the blood [17]. Hence, this study aimed to investigate whether an association exists between gingival pigmentation and skin color and its correlation with serum ferritin levels in thalassemia patients.

## Materials and methods

This prospective study involved 100 patients aged five to 50 years (62 males and 38 females) with β-TM visiting Thalassemia Day Care Centre, Ahmedabad, India. Informed consent was obtained from all patients enrolled. Parents gave consent in the case of child patients. The purpose of the study was explained to them in the regional language.

Subject selection

Subjects were selected according to inclusion and exclusion criteria set for the study. The sample size was set to 100 using consultation with the statistician. Subjects of all age groups were selected for the study without gender bias and with all maxillary and mandibular anterior teeth. Subjects having pathologic conditions that produce oral pigmentation such as Addison's disease, Albright syndrome, Peutz-Jeghers syndrome, and melanoma were excluded from the study. Subjects exposed to heavy metals, taking antimalarial drugs, patients with periodontitis, gingival pathology, or smoking habit were also not included in the study. This prospective study was done at Thalassemia Day Care Center, Ahmedabad, India, for six months.

Clinical data recording

Demographic details (age and sex), past medical history, history of blood transfusion, iron chelator use status, complete blood count (CBC), and serum ferritin levels were recorded for all the patients. Gingival pigmentation score and skin color were also noted.

Methodology

Patients diagnosed with thalassemia major were enrolled for the study, and those cases met the inclusion and exclusion criteria. Evaluation of gingival pigmentation and skin color was done. Gingival pigmentation score was recorded in maxillary and mandibular anterior teeth using Dummett's oral pigmentation index (DOPI) [18].

The scoring criteria were as follows. Score 0: no clinical pigmentation (pink-colored gingiva); score 1: mild clinical pigmentation (mild light brown color); score 2: moderate clinical pigmentation (medium brown or mixed pink and brown color); score 3: heavy clinical pigmentation (deep brown or bluish-black color).

**Figure 1:**
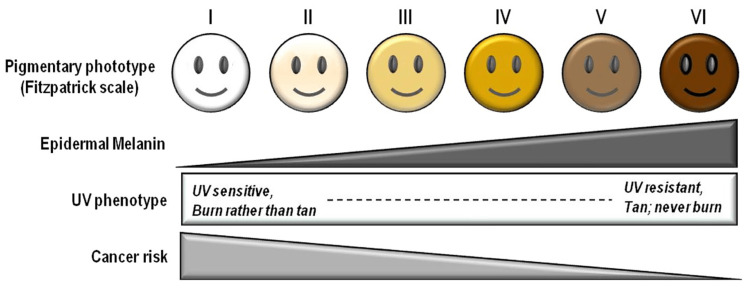
Fitzpatrick skin scale UV: ultraviolet.

**Figure 2:**
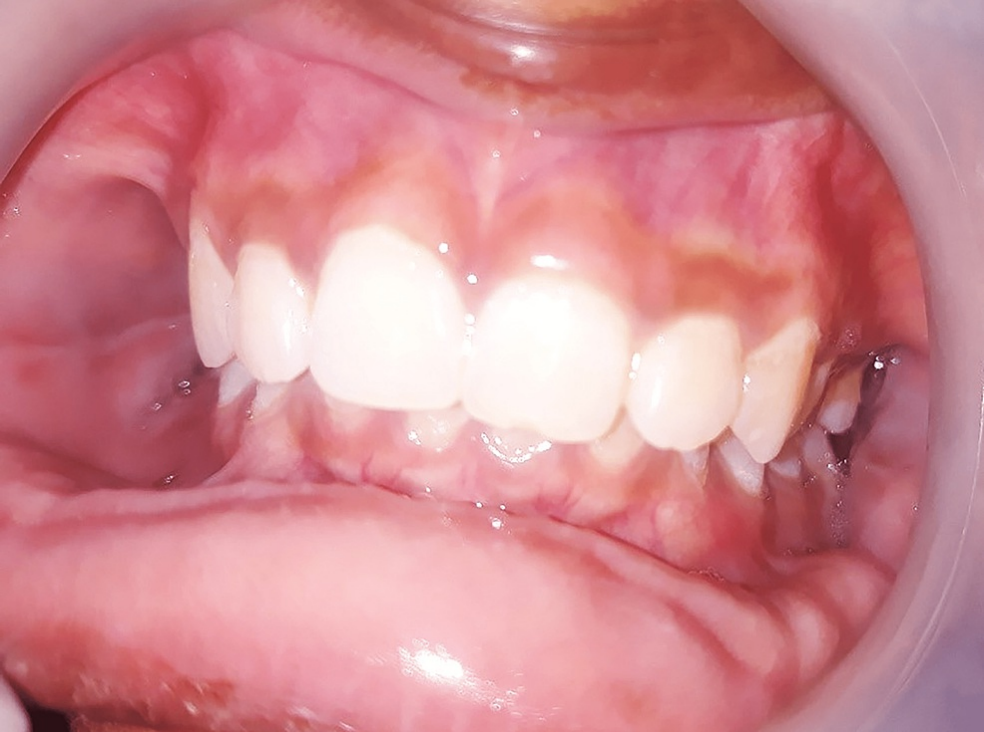
Gingival pigmentation with serum ferritin level of 1836 µg/ml, pigmentation score 1, and skin type 2

**Figure 3:**
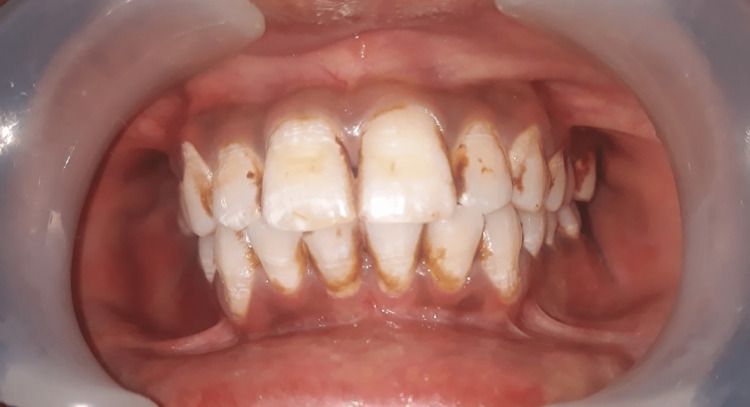
Gingival pigmentation with serum ferritin level of 2982 µg/ml, pigmentation score 2, and skin type 3

**Figure 4:**
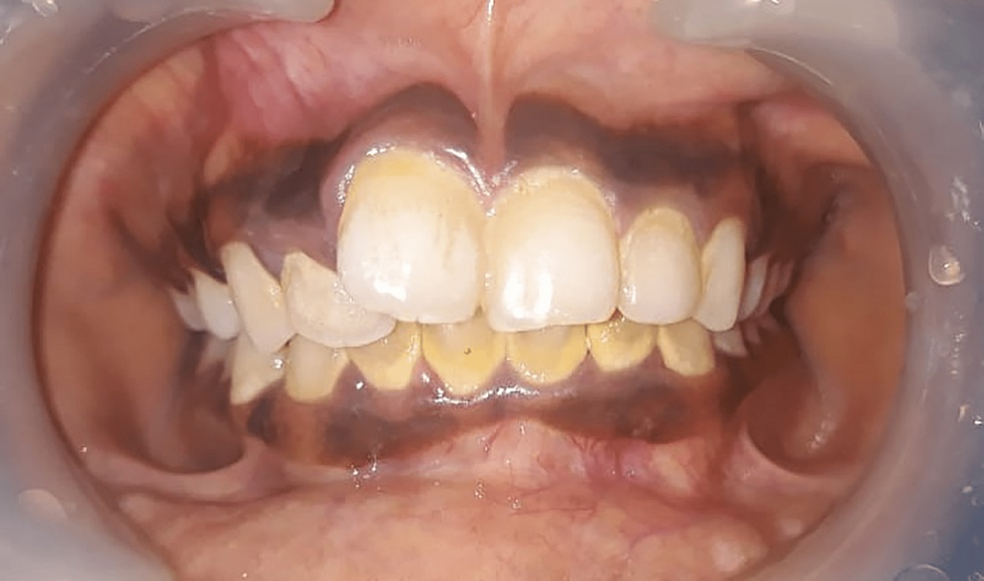
Gingival pigmentation with serum ferritin level of 6417 µg/ml, pigmentation score 3, and skin type 4

Skin color was inspected using the Fitzpatrick skin scale (Figure 1), as it is a recognized tool for dermatological research into human skin pigmentation [19]. It was recorded both on the region of the zygoma of the face and in a place that would not be exposed to the sun (behind the ear). The shade was evaluated in natural daylight against a neutral background. Different levels of serum ferritin and its co-relation with gingival pigmentation and skin color are illustrated in Figures 2-4.

Statistical analyses

The data were collected and transformed into tabular form. The data were then subjected to statistical analysis, which was done using Statistical Package for the Social Sciences (SPSS) version 20 (IBM Corp., Armonk, NY). The chi-square and Pearson correlation tests were compared and used to analyze the mean values. The probability value, i.e., p ≤ 0.05, was considered statistically significant.

## Results

**Figure 5:**
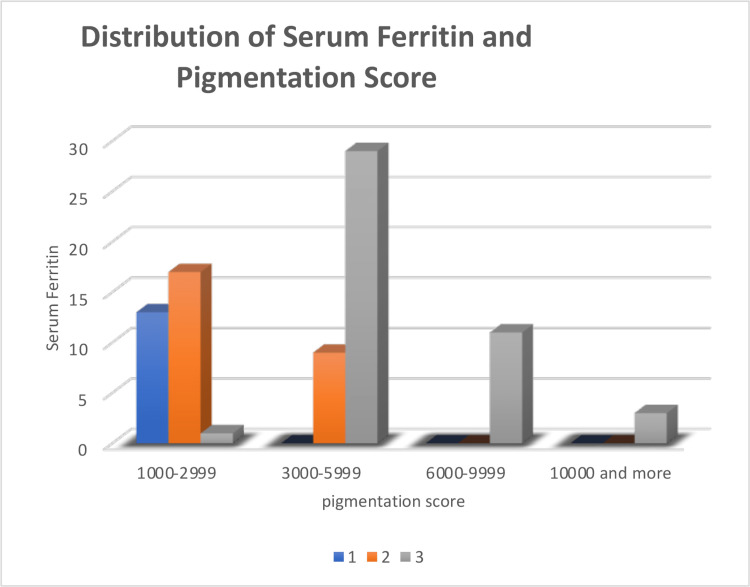
Distribution of serum ferritin and pigmentation score

A total of 100 patients were included in the study with an age range of five to 50 years (62 males and 38 females) with β-TM. The mean age of the patients was 17.6 ± 6.7 years (Table 1). All patients were using iron chelators as per the instructions by the hematologist. Analysis of serum ferritin levels and pigmentation scores showed that serum ferritin level was higher in patients with higher pigmentation scores (Figure 5). The co-relation (p = 0.001) between serum ferritin and gingival pigmentation was also found to be highly significant (Table 2).

**Table 1:** Demographic variables of the study

Demographic variables		Patients, n (%)
Gender	Male	62 (62)
	Female	38 (38)
Age range (years)	5-15	41 (41)
	16-25	44 (44)
	26-35	15 (15)

**Table 2:** Analysis of co-relation between gingival pigmentation and serum ferritin values

Variable	Pearson correlation value (r)	P-value
Serum ferritin and pigmentation score	0.752	0.001

**Figure 6:**
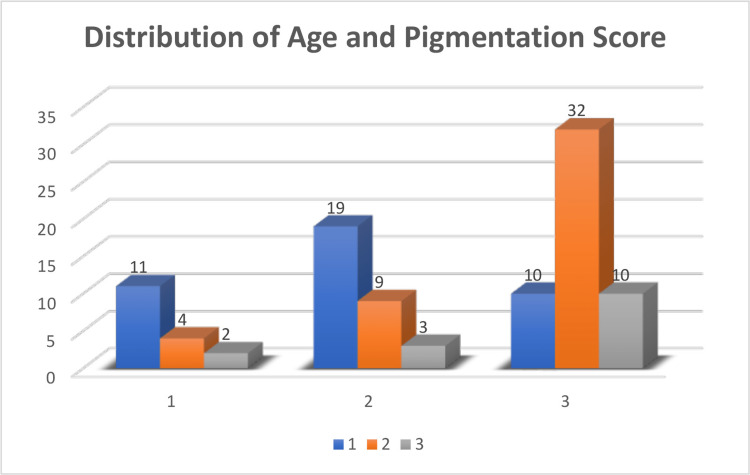
Distribution of age and pigmentation score

**Figure 7:**
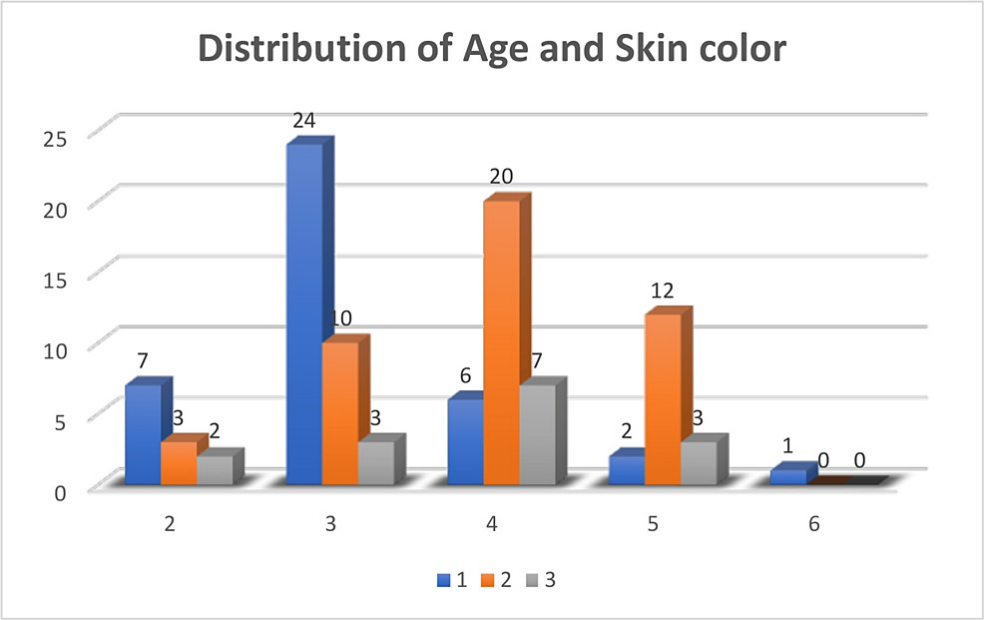
Distribution of age and skin color

Analysis of serum ferritin levels and skin color describes that high serum ferritin levels were observed with darker skin color (Table 3). A highly significant and positive correlation was found between serum ferritin and skin color (p = 0.001) (Table 4). Analysis of age and pigmentation score is depicted in Figure 6. It describes that with increasing age, gingival pigmentation increases. Similarly, the correlation between age and skin color is shown in Figure 7, which illustrates that skin color also darkened with age.

**Table 3:** Correlation of serum ferritin and skin color score by using Pearson correlation

Variable	Pearson correlation value (r)	P-value
Serum ferritin and skin color	0.827	0.001

**Table 4:** Distribution of serum ferritin and skin color SPSS software was utilized to analyze the data.

Variable	Skin color					P-value	
	2	3	4	5	6	0.001	
Serum ferritin							
1,000 to 2,999 ng/ml	8 (25.8)	20 (64.5)	3 (9.6)	0	0		
3,000 to 5,999 ng/ml	0	9 (23.6)	23 (60.5)	6 (15.7)	0		
6,000 to 9,999 ng/ml	0	0	4 (36.3)	7 (63.6)	0		
More than 10,000 ng/ml	0	0	0	2 (66.6)	1 (33.3)		

## Discussion

Thalassemia is a widespread genetic disorder in the Indian subcontinent [20]. β-TM affects approximately 1.5% of the world population [8,21,22]. It results from the defect in β-globin chain synthesis, leading to anemia. Hence, β-TM requires frequent transfusions of erythrocytes [23]. This blood transfusion at regular intervals has improved the longevity of life for thalassemia patients, and iron overload is an inescapable complication faced by thalassemia major patients [24]. These frequent blood transfusions may expose patients to other types of infections through the blood products.

Iron overloading is the most common complication of β-TM. Due to low hepcidin levels, duodenal iron level increases resulting in a high iron deposition. Also, iron gets deposited due to frequent erythrocyte transfusions [25,26]. Iron deposition in vital organs through the generation of reactive oxygen species (ROS) is a significant cause of morbidity and mortality, especially among elderly patients [27]. Therefore, quantitative, non-invasive methods for measuring body iron are needed that are safe, accurate, and readily available, and serum ferritin measurement is one of them. Serum ferritin's typical values for males and females are 12-300 ng/mL and 12-150 ng/mL, respectively [28].

Hattab reported that persons who have thalassemia have a higher rate of dental caries as compared to non-thalassemia patients [29]. The gum lining of the thalassemic patients becomes pale due to anemia. They might also experience a burning tongue due to folate deficiency [30]. Iron accumulates in the skin and mucous membrane, thereby getting darker in β-TM patients [31]. Youssry et al. showed that skin iron levels (with skin biopsy and atomic absorption spectrophotometry) were significantly correlated with hepatic iron levels in a patient with β-TM [31]. Spectrometry was used to confirm high iron deposition in the skin of patients with β-TM [32]. Bucak et al. also revealed significant findings between the visual skin color chart (VSCC) and the iron parameters of β-TM patients [14].

Various dermatological studies have shown that multiple charts can be used to evaluate skin and mucous color. These charts assess skin reactions and responses to dermatological treatments in injuries [33]. Similar to skin, the iron gets deposited beneath the gingiva, giving them different colors depending upon the amount of iron deposition at various stages of β-thalassemia.

Gingival pigmentation is associated with systemic diseases and syndromes like Addison's disease, Albright's syndrome, Basilar melanosis, β-thalassemia, mucocutaneous lesions, lichen planus, pemphigus, pemphigoid, pyogenic granuloma/granulomatous epulis, and other familial hamartoma syndromes [34]. Hyperpigmentation was found in 48.7% of study subjects in a study conducted in Iraq [35].

One more study stated that the cause of hyperpigmentation might be due to cutaneous iron deposition, which enhances melanin production [36]. Also, a study revealed that dermatological changes and oral mucosal pigmentation were seen in 17.9% of subjects and precluded dermatological manifestations among older multi- transfused β-TM patients [20]. Hence, gingival pigmentation score and skin color type can be used as a non-invasive tool to prevent the patients from invasive techniques and multiple needle prick injuries to investigate serum ferritin levels.

Limitations of the study

To the best of the authors' knowledge, this is the first study to evaluate and co-relate the gingival pigmentation with body iron levels in β-TM patients using DOPI. However, we must investigate histopathological changes in gingiva with excessive iron deposition. Further studies with greater sample size and other findings of β-TM patients, such as the size of the liver and spleen, should be compared to co-relate it with oral manifestations.

## Conclusions

This research can conclude that gingival hyperpigmentation and skin color changes have been seen among older multi-transfused β-TM patients. There is a significant correlation between serum ferritin levels and gingival hyperpigmentation in β-TM patients. There exists a definite relation between serum ferritin levels and skin color changes. There is a significant correlation between skin color and gingival pigmentation in β-TM patients. Further study with larger sample size is needed.
